# Supplementary material for: Locus coeruleus modulation of prefrontal dynamics during attentional switching in mice
Source: eLife. 2026 May 13;14:RP105911. doi: 10.7554/eLife.105911 (PMC13171103; doi:10.7554/eLife.105911)
Supplement: Supplementary file 1. [file elife-105911-supp1.docx]

|  | **Control** | | | | **Test** | | | |
| --- | --- | --- | --- | --- | --- | --- | --- | --- |
|  | C1 | C2 | C3 | C4 | T1 | T2 | T3 | T4 |
| Choice | 19/153 | 0/260 | 35/156 | 5/24 | 18/74 | 25/240 | 10/48 | 19/70 |
| History | 11/153 | 0/260 | 23/156 | 0/24 | 15/74 | 25/240 | 5/48 | 10/70 |
| Switch | 29/153 | 31/260 | 32/156 | 10/24 | 23/74 | 48/240 | 15/48 | 20/70 |
| All | 50/153 | 31/260 | 68/156 | 10/24 | 40/74 | 78/240 | 19/48 | 35/70 |
| Mixed | 9/50 | 0/31 | 18/68 | 4/10 | 15/40 | 19/78 | 9/19 | 12/35 |

**Supp. File 1**

The fraction of specific groups of task-encoding neurons in individual mice from the control (n = 4) and test (n = 4) groups.
